# Filtering Facepiece Respirators for Healthcare Workers Protection in the Time of COVID-19 Pandemic

**DOI:** 10.21315/mjms2021.28.3.14

**Published:** 2021-06-30

**Authors:** Chee Tao Chang, Philip Rajan, Victor Chee Wai Hoe

**Affiliations:** 1 Clinical Research Centre, Hospital Raja Permaisuri Bainun, Ipoh, Perak, Malaysia; 2 Centre for Epidemiology and Evidence-based Practice, Department of Social and Preventive Medicine, Faculty of Medicine, Universiti Malaya, Kuala Lumpur, Malaysia; 3 Occupational Safety Health and Environmental Unit, Universiti Malaya Medical Centre, Kuala Lumpur, Malaysia

**Keywords:** N95 respirators, healthcare workers, COVID-19, personal protective equipment, occupational health

## Abstract

Protecting healthcare workers (HCWs) who are in the frontline during the time of the COVID-19 pandemic is paramount. The filtering facepiece respirators (FFRs) or N95 respirator is one of the personal protective equipment (PPE) used to protect HCWs exposed to airborne pathogens in clinical practice or when performing aerosol generating procedures. The FFRs should be able to serve the intended purpose without causing additional health and safety hazards for the HCWs. The following commentary will provide some basic information on selecting correct FFRs and conducting fitness test.

## Editorial

On 11 March 2020, with an increase in the number of cases of the novel 2019 coronavirus disease (COVID-19) outside of China by 13-fold, the World Health Organization (WHO) declared COVID-19 as a pandemic (1). The number of COVID-19 cases worldwide continue to rise, and this has created an acute shortage of personal protective equipment (PPE), particularly the filtering facepiece respirators (FFRs), globally and also in Malaysia. The FFRs are a class of PPE used to protect the wearer from the exposure to environmental particulate matters. The N95 respirator is a FFR that meets the United States National Institute for Occupational Safety and Health (NIOSH), Center for Disease Control (CDC) and the United States of America N95 classification of air filtration (2).

The N95 respirators was originally used in industries. The first respirator certification programme was initiated by the United States Bureau of Mines back in 1919 and on 15 January 1920, they certified the first respirator (3). However, mask has been used for the prevention of infections since the time of the Black Death in the 17th Centuries in Milan and Rome (4). A study on the use of cloth masks among healthcare workers (HCWs) in 1918 found low rate of infection among those who used the masks (5).

A systematic review and meta-analysis of randomised trials comparing the use of medical mask versus N95 respirator for preventing COVID-19 in HCWs found that medical masks and N95 respirators offer similar protection against viral respiratory infection including coronavirus in HCWs during non-aerosol-generating care (6). The N95 respirator is recommended for HCWs performing aerosol generating procedures (AGP). The Ministry of Health Malaysia recommends the use of N95 for several procedures, including during ambulance transportation, providing care for COVID-19 patients who are not wearing mask, intubation and extubation of patients, and conducting oropharyngeal or nasopharyngeal swab (7).

The N95 respirator can filter at least 95% of airborne particles that are 0.3 microns in size or larger. The letter ‘N’ signifies that the FFRs can only be used in non-oil environment. If a FFR is to be used in an oily environment, then it should either have the letter ‘R’ — resistant to oil or ‘P’ — oil proof (2). Since all healthcare settings are considered non-oil environment, respirators with the ‘N’ designation offer sufficient protections. The list of the approved N95 respirators can be found on the NIOSH-Approved N95 Particulate Filtering Facepiece Respirators webpage. (https://www.cdc.gov/niosh/npptl/topics/respirators/disp_part/respsource3surgicaln95.html)

Other countries or agencies use different standards to certify FFRs. The followings are standards that are comparable to the NIOSH CDC N95 respirator standards: FFP2 (Europe EN 149-2001), KN95 (China GB2626-2006), P2 (Australia/New Zealand AS/NZA 1716:2012), Korea 1st class (Korea KMOEL-2017-64) and DS2 (Japan JMHLW-Notification 214, 2018).

The particulate-FFR that meets the above standards can be used in all clinical areas, however, the following criteria needs to be fulfilled:

FFRs with a valve should not be used in clinical areas, especially in areas where sterility is an important criterion. This is because the FFRs with a valve is designed for easy breathing, during the expiration phase. The use of FFRs in a sterile area will contaminated the area from the air exhaled by the user. If FFRs with a valve needs to be used in a sterile area, then it should be used in combination with a medical mask. The medical mask must be worn over the FFRs with a valve.

HCW using personal protective equipment (PPE) should ensure that the PPE is able to serve the intended purpose and does not cause additional health and safety hazards. In the case of the FFRs, HCW must ensure that the FFRs are fit for use as different FFRs have different design and size, all FFRs may not fit all HCW.

The FFRs should not hinder the breathing of the HCW. If the HCW finds that when using the FFRs, there is difficulty in breathing or the HCW fells breathlessness, then the HCW should replace the FFRs with new FFRs. The removal of the FFRs should be carried out in accordance with proper doffing procedures. For HCW who have cardiac or respiratory conditions; they should seek further medical assessment before the use of FFRs.

To ensure that the FFRs fit the HCW, fit-test and fit-check should be performed:

Fit-test should be performed for all HCW for any new type of FFRs. Fit test can be qualitative fit-test-sensory detection of a test agent such as taste, smell; or quantitative fit tests-uses an using a non-hazardous test aerosol generated in a test chamber and employ instrumentation to quantify the fit of the respirator (8).

Fit-Check: HCW are required to pick the most suitable FFRs from the available number of models and sizes, and worn at least five minutes to assess the following criteria: i) comfort-position of mask on the nose, room for eye protection, room to talk, position of mask on face and cheek; ii) adequacy of the respirator fit-chin properly placed, optimum strap tension, fit across nose bridge, proper distance from nose to chin, tendency of the respirator to slip, self-observation in mirror to evaluate fit and respirator position; iii) seal check recommended by the respirator manufacturer; and iv) test exercises-normal breathing, deep breathing, turning head side to side, moving head up and down, talking (8).

Due to overwhelming demand of masks, there may be a need to conserve the disposable ones. The CDC advices engineering and administrative controls, prioritising usage and alternatives where feasible. In situations of extreme shortage, extended use (serial use for multiple patients over a certain time period) or re-use (removing and redonning) of disposable may be an option (9).

However, the risks of extended use and re-use are unknown. There is a risk of contact transmission by touching a contaminated surface of the respirator and subsequently touching mucous membranes of the face. Therefore, this strategy is not to be considered for high risk procedures such as aerosol generating procedures and the N95 respirator should be disposed when contaminated by blood, secretions and bodily fluids of patients. When practiced, consider using a face shield over the disposable N95 respirator and/or hand hygiene before and after touching the respirator (9). When a disposable respirator is considered for re-use, suggested methods for decontamination include hydrogen peroxide vaporisation, microwave-generated steaming, and dry heating, and moist heating, and ultraviolet C irradiation (9–12). Nevertheless, disposal of respirators after single use is recommended under ordinary situation.

The FFRs is a critical component of adequate personal protective gear among HCWs when dealing with COVID-19 patients. Understanding how the FFRs work and the proper usage is important in providing the expected levels of protection for HCWs.
